# Diversifying the health workforce: a mixed methods analysis of an employment integration strategy

**DOI:** 10.1186/s12960-021-00606-y

**Published:** 2021-05-05

**Authors:** Andrea Baumann, Mary Crea-Arsenio, Dana Ross, Jennifer Blythe

**Affiliations:** 1 Global Health Office, Faculty of Health Sciences, McMaster University, 1280 Main Street West, MDCL 3500, Hamilton, ON L8S 4K1 Canada; 2 School of Nursing, McMaster University, 1280 Main Street West, MDCL 3500, Hamilton, ON L8S 4K1 Canada

**Keywords:** Canada, COVID-19, Employment, Immigration, Nurses, Workforce diversity

## Abstract

**Background:**

Historically, immigration has been a significant population driver in Canada. In October 2020, immigration targets were raised to an unprecedented level to support economic recovery in response to COVID-19. In addition to the economic impact on Canada, the pandemic has created extraordinary challenges for the health sector and heightened the demand for healthcare professionals. It is therefore imperative to accelerate commensurate employment of internationally educated nurses (IENs) to strengthen and sustain the health workforce and provide care for an increasingly diverse population. This study aimed to determine the effectiveness of a project to help job-ready IENs in Ontario, Canada, overcome the hurdle of employment by matching them with healthcare employers that had available nursing positions.

**Methods:**

A mixed methods design was used. Interviews were held with IENs seeking employment in the health sector. Secondary analysis was conducted of a job bank database between September 1 and November 30, 2019 to identify healthcare employers with the highest number of postings. Data obtained from the 2016 Canadian Census were used to create demographic profiles mapping the number and proportion of immigrants living in the communities served by these employers. The project team met with senior executives responsible for hiring and managing nurses for these employers. The executives were given the appropriate community immigrant demographic profile, a manual of strategic practices for hiring and integrating IENs, and the résumés and bios of IENs whose skills and experience matched the jobs posted.

**Results:**

In total, 112 IENs were assessed for eligibility and 95 met the inclusion criteria. Twenty-one healthcare employers were identified, and the project team met with 54 senior executives representing these employers. Ninety-five IENs were subsequently matched with an employer.

**Conclusions:**

The project was successful in matching job-ready IENs with healthcare employers and increasing employer awareness of IENs' abilities and competencies, changing demographics, and the benefits of workforce diversity. The targeted activities implemented to support the project goal are applicable to sectors beyond healthcare. Future research should explore the long-term impact of accelerated employment integration of internationally educated professionals and approaches used by other countries.

## Background

In April 2020, the population of Canada surpassed 37.9 million [1]. Between January and March 2020, 82% of population growth nationwide was due to international migration [1]. There were more than 64,000 refugee claimants in 2019, the highest number on record [2]. Despite COVID-19, the Canadian government remains committed to immigration. The 2021‒2023 Immigration Levels Plan shows immigration targets have been increased to an unprecedented level of more than 1.2 million newcomers over the next 3 years to support economic recovery in response to the pandemic and encourage long-term economic growth [3]. The largest share of newcomers (60%) will be from the Economic Class, which includes highly skilled applicants with foreign work experience relevant to the Canadian labor market [3].

Ontario is Canada's most populous province and the top destination for immigrants, particularly the Greater Toronto Area (GTA). As of April 2020, more than 14.7 million people and more than 46% of all immigrants in Canada were living in Ontario [4]. In December 2019, the province petitioned the federal government to raise the number of economic immigrants to Ontario to more than 13,000 over the next 2 years [5]. As population demographics change, healthcare organizations must change to meet the needs of increasingly diverse patients. Likewise, as immigration continues, commensurate employment of highly skilled newcomers must be accelerated.

Internationally educated nurses (IENs) can play an important role in the provision of care for the changing population. The literature demonstrates that health inequities for newcomers are reduced when health workforce diversity is increased [6, 7]. In 2019, IENs accounted for 9% of the regulated nursing supply in Canada [8]. However, they face significant challenges obtaining employment equivalent to their skills and experience. For example, registration requirements, employer bias, language and communication, insufficient support, and unfamiliarity with interview practices and résumé preparation [9–11].

Employment in nursing fluctuates due to the development of new healthcare facilities, retirement rates of the existing workforce, changes in healthcare policy and delivery models, funding decisions, and economic conditions [12–14]. COVID-19 has increased the need for nurses but has not necessarily increased the likelihood of internationally educated applicants being hired. The majority (82%) of highly skilled immigrant women in a recent survey indicated the pandemic has had a detrimental effect on their employment and career paths [15].

**Fig. 1:**
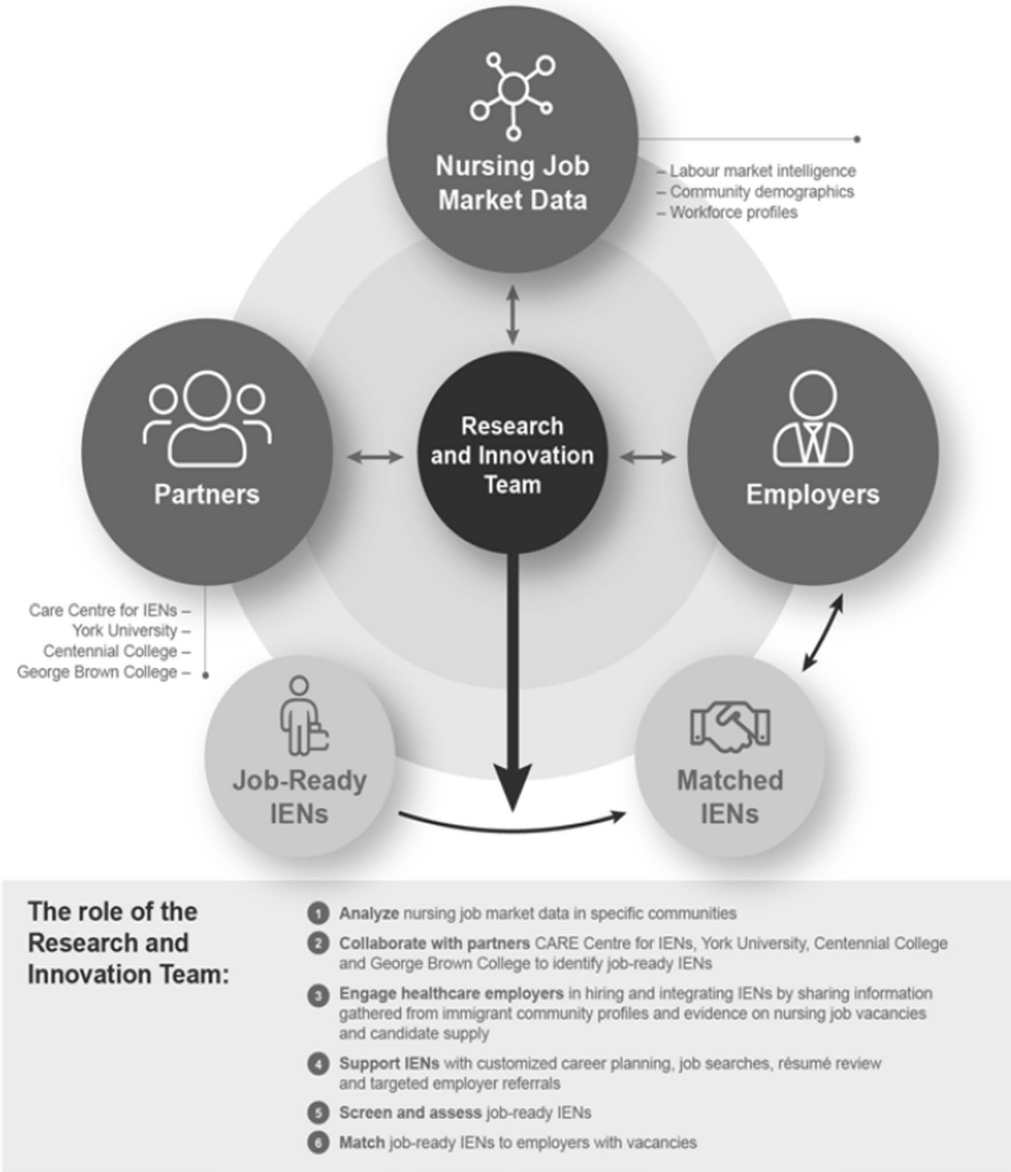
Project schema: overcoming the hurdle of employment for internationally educated nurses (IENs)

The Panel on Employment Challenges of New Canadians found that "employers' risk-averse nature . . . causes them to shy away from hiring immigrants" [16], p 11]. Consequently, many internationally educated professionals settle for low-paying survival jobs outside their profession [17, 18]. As a result, their occupational identity and earning potential are lost and a valuable resource is left untapped. In 2018, a project was piloted to help job-ready IENs in Ontario overcome the hurdle of employment by matching them with healthcare employers that had available nursing positions. As shown in Figure 1, this is a complex process with many interrelated elements.

The project builds on seminal work that included the creation of an online repository of information for healthcare employers and a manual of evidence-informed strategic practices for hiring and integrating IENs [11, 19]. Targeted activities were implemented to meet the project goal: engaging healthcare employers, analyzing a job bank database, developing community immigrant demographic profiles and workforce profiles, communicating with job-ready IENs and senior executives, and collaborating with partners. The purpose of our study was to assess the effectiveness of the project. The findings have significant implications for healthcare delivery and health human resources and can inform policies and procedures to meet the needs of diverse patients and increase commensurate employment of IENs.

## Methods

### Design and participants

A mixed methods design was used. Internationally educated nurses were recruited from various partners, including a university program for IENs, community colleges that provide bridge training programs, and a provincial non-government settlement organization. Bridge training programs offer rapid access to skills upgrading for new immigrants and are funded by the government of Ontario [20].

The project was advertised through program coordinators and case managers. To be eligible, IENs had to be landed immigrants who were registered with the College of Nurses of Ontario (CNO) and seeking employment as a nurse. The project team conducted telephone interviews with IENs to determine their employment preferences, experience, and qualifications. The IENs were provided with customized career planning and information about the hiring protocols used by employers and the key words they look for in résumés.

Secondary analysis of a job bank database was conducted to validate the availability of nursing jobs in Ontario between September 1 and November 30, 2019 and to identify healthcare employers with the highest number of postings. Senior executives responsible for decision-making related to hiring and managing nurses were recruited from the employers, and 2016 Canadian Census data were used to create demographic profiles that mapped the number and proportion of immigrants living in the communities served by the employers. The project team initiated hour-long face-to-face onsite meetings with the senior executives during which they were given the appropriate demographic profile, a manual of strategic practices for hiring and integrating IENs, and the résumés and bios of IENs whose skills and experience matched the jobs posted.

### Data collection

Data collected from the IENs included nursing education, clinical experience, registration status, and work preferences. The senior executives provided data on the recruitment and hiring strategies of their employers, nursing vacancies, and challenges related to workforce planning and the provision of care for diverse populations.

### Analysis

The job bank database gathers information at the location, occupation, and sector levels from more than 25,000 sources, including job boards and corporate career sites. Search terms included "registered nurse" and "registered practical nurse". General membership data on domestically educated nurses (DENs) and IENs were obtained from the 2019 CNO registration database and compared with the geographic distribution of job postings.

The following measures were used to evaluate the effectiveness of the project: counts of the number of employers contacted, the number of senior executives who met with the project team, the number of IENs referred to employers, and the number subsequently hired. Other indicators included employer interest in workforce diversity and senior executives' experiences hiring IENs. The outcome measures were an increase in the number of employers hiring and integrating IENs and the number of IENs successfully matched with an employer.

## Results

In total, 112 IENs were assessed for eligibility and 95 (86%) met the inclusion criteria. Most were female (98%), 45 years of age or younger (92%), and living in the GTA (94%). The majority had immigrated from India and the Philippines (60%). Over half (58%) had entered Canada through the Federal Skilled Worker Program, 31% through the family class, and 9% through other classes such as student visas and the live-in caregiver program. All had immigrated between 2012 and 2018. On average, they had 5.9 years of nursing experience prior to entering Canada and 46% had worked as a nurse in at least one other country in addition to their home country. The majority had completed a bridge training program prior to registration with the CNO (Table 1).

**Table 1:** Number of internationally educated nurses who completed a bridging program

Bridging program completed	Total number	Percentage of total
Yes	70	74%
No	23	24%
Unknown	2	2%
Total	95	100%

Source: McMaster University, unpublished program data

Point-in-time data were obtained for 5518 nursing positions across the province. Thirty-eight percent were full-time positions, 30% were part-time positions, 21% were casual/temporary positions, and another 10% were unknown or posted as multiple positions. Over 60% of the positions were in the hospital sector, followed by 22% in the community sector, and 8% in long-term care. Twenty-one healthcare employers posted 81% of the positions: 17 hospital corporations, 3 long-term care homes, and 1 community healthcare organization. Together they operate 163 sites across Ontario. The project team met with 54 senior executives representing these employers. The executives included nursing leaders, human resource directors, and vice-presidents of clinical care. They were given the appropriate immigrant demographic profile and the résumés and bios of job-ready IENs. Table 2 shows the number of IENs who were subsequently matched with an employer and the sectors into which they were hired.

**Table 2:** Employment matches by sector for internationally educated nurses

Sector	Total number matched	Percentage of total
Hospital	57	60%
Long-term care home	21	22%
Home care	6	6%
Teaching	1	1%
Unknown (e.g., blanks)	3	3%
Other (e.g., community care)	4	4%
Multiple sector(s)	3	3%
Total	95	100%

**Fig. 2:**
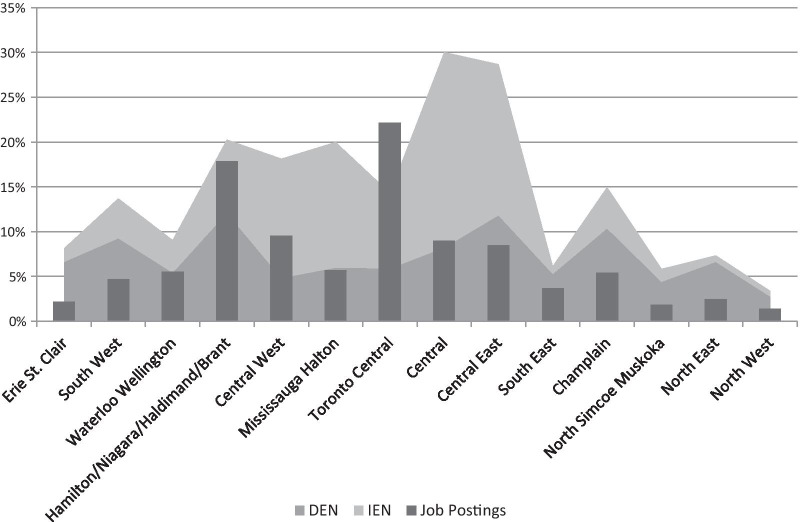
Distribution of job postings and place of residence for domestically educated nurses (DENs) and internationally educated nurses (IENs) across Ontario, Canada. Source: TalentNeuron database of job postings for registered nurses and registered practical nurses and College of Nurses of Ontario registration database 2019

Based on the most recent Canada Census data, 39% of immigrants nationwide lived in Ontario in 2016 and immigrants accounted for 46% of the population in the City of Toronto [21, 22]. The proportion of non-official language speakers ranged from 27% to 62% in the GTA [23]. The jobs posted by the employers were concentrated in the GTA and the southwestern region of the province. However, compared to DENs, there was a higher number of IENs residing in the central urban regions of the province. Figure 2 shows the distribution of job postings and residential locations of IENs and DENs across Ontario.

## Discussion

Overcoming the hurdle of employment is a fundamental step in immigrant integration. Employment supports current and future financial stability, quality of life, and personal and family security. It also helps immigrants connect with their communities, establish networks, and contribute to the economy [24, 25]. Employment is particularly important for female newcomers as they earn less and experience greater difficulties obtaining jobs than male newcomers do [26]. Internationally educated nurses are predominantly female and account for a substantial number of highly skilled immigrants to Canada [27]. We evaluated the effectiveness of a project intended to accelerate their uptake into the Ontario healthcare workforce by matching them with employers that had available nursing positions. Based on the results, the strategy was successful.

The majority of IENs had entered Canada under the Federal Skilled Worker Program in which immigrants are selected based on their skills and ability to contribute to the national economy. The program is managed through the online Express Entry system intended to expedite processing of immigrant applications [28]. Nonetheless, once they arrive in Canada many highly skilled newcomers find themselves in a holding pattern for suitable employment even when their profession is in demand. It is estimated that immigrants in Canada experience wage losses of more than $12 billion annually because of employment challenges [29]. Covell et al. found that IENs with some professional experience obtained in Canada were more likely to find employment [27]. However, highly skilled newcomers have expressed concern that Canadian experience is "a coded way for employers to favour the Canadian-born" [30], p 6].

It is important to acknowledge that hiring practices and requirements can be mechanisms of discrimination. Employment decisions are frequently made across departments and with input from a panel, but there are typically arbiters who have the ultimate say. Our previous work (i.e., online repository of information for healthcare employers and employer manual) highlighted the need to identify and target key decision-makers in healthcare organizations in order to increase their willingness to hire IENs. This was achieved in the face-to-face meetings with the senior executives.

Internationally educated nurses in Canada and other countries have reported covert and overt prejudice when applying for nursing positions and in the work environment [31, 32]. The literature reflects that progress in equitable employment has remained slow and fraught with barriers [33, 34]. The targeted activities implemented to meet the project goal helped address some of these barriers: lack of employer understanding, time constraints, hiring bias, and scarcity of data.

The IENs were current members of the CNO and met all registration requirements, e.g., demonstrated educational equivalency and recency of practice. Giving senior executives their bios and résumés made them aware that the IENs had the abilities and competencies to perform their roles safely and ethically. This awareness is vital for helping IENs enter the nursing job market. The senior executives acknowledged the need for greater "exposure" to IENs and employer understanding that "international experience is just as good as in Canada".

Those responsible for hiring and managing nurses frequently have busy schedules and limited time to sift through applications to determine which candidates are qualified. Prior to meeting with the senior executives, the project team reviewed the IENs' bios and résumés and provided editorial assistance. That a significant number of IENs did not already have job-ready résumés reinforces their need for support, particularly given that senior executives stressed the importance of accurate and complete applicant information when making hiring decisions.

The literature shows that immigrant and minority job applicants are often overlooked in favor of native-born applicants [35, 36]. Researchers from one study cited insufficient information during the hiring phase as a possible reason [37]. Other studies have found the work experience, education, and skills of newcomers are undervalued, ignored, or improperly assessed by those in charge of hiring [38, 39]. Our study reinforces that readily accessible comprehensive data, including suitability of fit between the skills of IEN applicants and the requirements of employers, contributes to more objective hiring. Moreover, it can assist the employment efforts of IENs. When IENs know which organizations are hiring and which positions they are looking to fill, they can target employers within their preferred geographic areas and jobs within their scopes of expertise.

Analysis of the job bank database proved there are jobs in the health sector and the demand for nurses is on the rise. Analysis of the distribution of DENs, IENs, and job postings identified employment locations across Ontario and indicated there was a supply of job-ready IENs. The community immigrant demographic profiles emphasized the necessity for workforce diversity, but composition of the health workforce in communities with significant and increasing immigrant populations was suboptimal.

This issue is not unique to Canada, as the argument for greater diversity in the health workforce has been made in Australia, Europe, and the United States [40–42]. Providing employers with evidence that communities are changing fosters recognition that the focus of hiring must shift from meeting only immediate needs and service requirements and consider population requirements as well. Aversion to making this shift contributes to health inequities for newcomers and increases their risk of falling through gaps in the delivery of care. It also reinforces the wage gap between DENs and IENs and contributes to disparities in employment and working conditions for the latter.

Building a health workforce that reflects the communities it serves can improve health literacy for immigrants and enhance their experiences and health outcomes [43, 44]. Furthermore, it can increase the economic contributions and gains of a highly skilled cadre of newcomers. By hiring IENs, the employers strengthened the capacity of their organizations to serve a broad spectrum of people and boosted the earning power of IENs. They also increased the potential for enriched organizational culture and greater cultural competence as IENs have unique experiences from which others can learn.

To assist IENs in overcoming the hurdle of employment, strong relationships between bridge training programs, immigration and settlement organizations, and employers are paramount. Additionally, healthcare organizations must value and model diversity in their labor practices and internal culture. Human resource departments should have an Office of Diversity that has input into recruitment and staffing and can tap into hiring initiatives for job-ready IENs. It is also recommended that employers commit to workplace equity and inclusion. For example, by recognizing and capitalizing on the ability of IENs to provide professional care across the organization. Equitable workforce participation of IENs benefits them, the organization, and patients. Real and substantive changes that disrupt the status quo are imperative such as professional development opportunities for IENs equal to those of DENs, encouraging and endorsing the advancement of IENs as nurse leaders, and including them in policymaking and decision-making. Enabling IENs to excel and positioning them to do so can have impacts at the local, provincial, and national levels.

The population is changing and not only in major urban centers. A diverse health workforce can help ensure health systems and organizations are equipped to respond. In addition to driving population growth, immigration has a significant effect on economic growth. Given the Canadian government's plan to increase immigration, labor market diversification and accelerated hiring and integration of highly skilled newcomers should be prioritized in policy and practice. Mounting concerns about nursing shortages due to COVID-19 makes this issue even more urgent [45, 46].

### Limitations

The job bank database does not reflect all vacancies in Ontario as many healthcare employers rely on internal applicants rather than posting externally. Even so, analysis of the database does provide salient information on the nursing labor market at a set point in time and establishes a baseline.

## Conclusion

This study demonstrates that understanding population dynamics is important for strategic workforce planning. As community demographics change, the composition of the healthcare workforce must change. This can be achieved via employment strategies that include job matching and demographic profiling. However, it is not enough that employers recognize the need for increased workforce diversity. They must also recognize the advantages for their organizations.

Results of our study have significant implications for healthcare delivery and health human resources and can inform policies and procedures to meet the needs of diverse patients and increase commensurate employment of IENs. The project schema provides a roadmap for labor market integration of newcomers, and the targeted activities implemented to support the project objective are applicable to sectors beyond healthcare. Future research should explore the long-term impact of accelerated employment integration of internationally educated professionals and approaches used by other countries.

## Data Availability

The datasets used and/or analyzed during the current study are available from the corresponding author on reasonable request.
